# Birth Outcomes of Latin Americans in Two Countries with Contrasting Immigration Admission Policies: Canada and Spain

**DOI:** 10.1371/journal.pone.0136308

**Published:** 2015-08-26

**Authors:** Marcelo L. Urquia, Zoua M. Vang, Francisco Bolumar

**Affiliations:** 1 Centre for Research on Inner City Health, Li Ka Shing Knowledge Institute, Keenan Research Centre, St Michael’s Hospital, Toronto, Canada; 2 Dalla Lana Faculty of Public Health, University of Toronto, Toronto, Canada; 3 Institute for Clinical Evaluative Sciences, Toronto, Canada; 4 Sociology Department, McGill University, Montreal, Quebec, Canada; 5 CIBERESP and Universidad de Alcalá, Madrid, Spain; 6 City University of New York, School of Public Health at Hunter College, New York, New York, United States of America; Public Health Agency of Barcelona, SPAIN

## Abstract

**Background:**

We delved into the selective migration hypothesis on health by comparing birth outcomes of Latin American immigrants giving birth in two receiving countries with dissimilar immigration admission policies: Canada and Spain. We hypothesized that a stronger immigrant selection in Canada will reflect more favourable outcomes among Latin Americans giving birth in Canada than among their counterparts giving birth in Spain.

**Materials and Methods:**

We conducted a cross-sectional bi-national comparative study. We analyzed birth data of singleton infants born in Canada (2000–2005) (N = 31,767) and Spain (1998–2007) (N = 150,405) to mothers born in Spanish-speaking Latin American countries. We compared mean birthweight at 37–41 weeks gestation, and low birthweight and preterm birth rates between Latin American immigrants to Canada vs. Spain. Regression analysis for aggregate data was used to obtain Odds Ratios and Mean birthweight differences adjusted for infant sex, maternal age, parity, marital status, and father born in same source country.

**Results:**

Latin American women in Canada had heavier newborns than their same-country counterparts giving birth in Spain, overall [adjusted mean birthweight difference: 101 grams; 95% confidence interval (CI): 98, 104], and within each maternal country of origin. Latin American women in Canada had fewer low birthweight and preterm infants than those giving birth in Spain [adjusted Odds Ratio: 0.88; 95% CI: 0.82, 0.94 for low birthweight, and 0.88; 95% CI: 0.84, 0.93 for preterm birth, respectively].

**Conclusion:**

Latin American immigrant women had better birth outcomes in Canada than in Spain, suggesting a more selective migration in Canada than in Spain.

## Introduction

The healthy migrant effect (also known as healthy migrant bias) refers to the phenomenon wherein immigrants are healthier than the local native-born population and their co-nationals who did not emigrate.[1] The health advantage of immigrants in industrialized countries has been observed despite their socioeconomic disadvantage and poorer access to healthcare relative to the local population; this observation has been regarded as a paradox (immigrant or Latino paradox).[1] Both the healthy migrant effect and the immigrant paradox are thought to stem from selective migration, that is, the result of processes that select (or self-select) healthier individuals for migration. Selective migration may be associated with health in at least two non-exclusive ways. First, healthy individuals may self-select themselves for migration on the basis of their perception of successfully overcoming the challenges and uncertainties of relocation compared to those with health problems, who may choose not to take risks and remain in their home countries.[2] Second, immigration policies in the receiving countries regulate admission of foreigners and pathways to permanent residence and citizenship. Hence, immigration policies may act as a second filter for those who self-selected themselves to immigrate, presumably creating a “cream skimming” effect and resulting in the admission of an even healthier group of immigrants.

The selective migration hypothesis has been examined most extensively in the context of adult health, and mostly among immigrants in the United States. Some of these studies found some support for the selective migration hypothesis[3–6] but others did not.[7,8] However, with some exceptions,[9–12] international selective migration has not been exhaustively tested in perinatal health research. Instead, most studies have examined the healthy migrant effect by comparing birth outcomes between foreign-born versus native-born women.[13–16] This approach has produced inconclusive evidence because the direction and magnitude of the associations depend on the baseline level of health observed in the native-born reference groups of the different receiving countries.[14] In this study we delve into the selection hypothesis from a different perspective; by comparing birth outcomes of one distinct group of emigrants (i.e., Latin Americans) residing in two countries with different immigration admission policies (Canada and Spain). We hypothesize that Canada’s more restricted immigration policy will result in stronger positive selection of immigrants than in Spain and therefore birth outcomes of Latin Americans giving birth in Canada will be more favourable than those of their same-country counterparts giving birth in Spain.

Immigration of Latin Americans to Canada and Spain provides an opportunity to study the influence of selective migration on health. Indeed, for many Latin Americans, immigrating to Canada has been more restricted than to Spain. As a traditional immigrant-receiving country Canada’s immigration regime has evolved since the country’s founding and currently most immigrants to Canada are selected on the basis of a point system that rewards characteristics associated with success in the labor market, such as working knowledge of English/French as a second language, education, work experience and wealth. Canada also admits lower human capital immigrants such as refugees and family-class migrants to a lesser extent.[17] Unauthorized stay, although existent, is not a significant source of immigration in Canada. As of September 2007, the Canada Border Service Agency determined that there were about 63,000 individuals with either enforceable removal orders or outstanding immigration warrants for removal, including 41,000 individuals whose whereabouts were unknown to the Agency.[18] These figures, although not negligible, represent a small proportion of the approximately 6.2 million foreign-born individuals living in Canada.[19] In contrast, Spain recently transitioned from being an emigration to an immigration country. Legal immigration dramatically increased in Spain from about 500 000 individuals in 1995 to about 4 million in 2007, 33% of whom were from Latin American countries.[20] Immigration to Spain subsided in the wake of the 2007–2008 global financial crisis. The number of refugees admitted to Spain is negligible.[20] However, unauthorized residence, which has been shown to attract lower human capital immigrants, is substantial with an estimated 1.2 million undocumented individuals in 2007.[21] The relatively large flow of unauthorized immigration in Spain was fueled by a robust demand for low-skill labor and structural deficiencies in the management of immigration.[22] Spain offered regularization programs to unauthorized immigrants in 2000/01 and 2005. Of the almost 700,000 applicants to the 2005 program, 39% of them were from Latin American countries and 21% from Ecuador alone.[22]

Latin Americans face fewer challenges to migrate to Spain than to Canada because of the common Spanish language and citizenship rights among those who have a Spanish ancestor up to two generations. Latin America has been the main destination of Spanish emigrants from the sixteenth century to the 1970’s. About 2.5 million Spanish emigrants headed towards Latin American countries during the 20^th^ century.[21] By 2006, 62% of the approximately 380,000 naturalized citizens originated from Latin America.[20]

Based on the marked differences between the admission contexts of the two countries, if selective migration is associated with better health we would expect more favourable outcomes among Latin Americans who migrated to Canada compared to those who migrated to Spain, as a result of Canada’s stricter selection and admission policies. Low birthweight and preterm birth are adverse pregnancy outcomes sensitive to environmental influences, such as social class.[23] They are strongly associated with infant mortality and morbidity and impaired health and wellbeing in adult life, having detrimental emotional and financial consequences for families and multi-sectorial public services.[24]

We delved into the selective migration hypothesis by comparing mean birthweight at term, low birthweight and preterm birth between Latin American immigrants giving birth in Canada vs. Spain, using comparable contemporary nationwide data.

## Materials and Methods

### Study design and population

This cross-sectional population-based comparative study uses national birth certificate data from Canada and Spain. The study population is composed of singleton liveborn infants in Canada (2000–2005) and Spain (1998–2007) to women born in Spanish-speaking Latin American countries and native-born women (Canadian-born women in Canada and Spanish-born women in Spain) in the last decade available in national birth certificate data. The longer study period for Spain reflects availability of data; however, the midpoint is the same for both countries, thus maintaining comparability. Both registers collect information on maternal country of birth and other sociodemographic characteristics. To minimize bias due to potential outliers, we only included records with birthweights within 250–5999 grams and gestational age within 20–45 weeks.

### Data

Information on birth outcomes in Canada was obtained from the linked live birth-death file of the Public Health Agency of Canada’s Canadian Perinatal Surveillance System. Approval for use of this data was obtained from the Agency and Statistics Canada. Ethics approval was not legally required since data were anonymized, analyses were conducted in restricted access Research Data Centers, and guidelines for release of results were adhered to. Likewise, this study only used anonymized Spanish birth certificate data compiled by the National Institute of Statistics and the review of an Ethics Research Committee was not legally required in Spain.

Secondary analyses were conducted with a subsample of Latin American women delivering in Ontario hospitals (1998–2007) and captured through a linkage between the official immigration register, the Citizenship and Immigration Canada database, and the Discharge Abstracts Database, which comprise all hospital births. Data collection methods and use of Ontario hospital data were approved by the St. Michael’s Hospital Research Ethics Board. These analyses were conducted at the Institute for Clinical Evaluative Sciences (ICES) in Toronto, Ontario. ICES is a prescribed entity under Section 45 of the Ontario Personal Health Information Protection Act (PHIPA) 2004. As such, it is permitted to receive (collect) and use personal health information without consent. ICES implements strict privacy and confidentiality practices and procedures that have been reviewed and approved by the Information and Privacy Commissioner of Ontario as required under the Act.[25]

Outcome measures were singleton mean birthweight at term (37–41 weeks gestation), low birthweight (< 2500 grams) and preterm birth (<37 weeks of gestation). In Canadian birth certificates, the information on gestational age is recorded by the attending physician, nurse or midwife. In Spain, it is recorded by the parents based on the information provided to them by the birth attendant. A Spanish validation study using records of a single hospital as the gold standard, found that the sensitivity and specificity of birth certificate data for low birthweight were 90% and 99%, respectively and 78% and 98% for preterm birth.[26] Most notably, the birthweight distributions exhibited an almost perfect overlap between the curves at 37 and higher gestation weeks.

*Maternal country of birth* included Canada, Spain, Argentina, Bolivia, Chile, Colombia, Cuba, Ecuador, El Salvador, Honduras, Mexico, Nicaragua, Paraguay, Peru, Uruguay, Venezuela and Rest of Latin America (Costa Rica, Dominican Republic, Guatemala and Panama). The latter category was created by collapsing countries contributing very few births in at least one of the two countries. *Country of delivery* included Canada and Spain.

### Analytic strategy

For descriptive statistics, we conducted a side-by-side analysis based on common definitions of the outcomes and birth characteristics in the two national datasets. Due to the restricted access of each dataset, it was not possible to pool the datasets of the two countries at the individual level but we could do so in aggregate form.[27] In order to compare birth outcomes in Canada versus Spain, we calculated within each dataset the mean term birthweight and the proportion of low birthweight and preterm birth, within strata of covariates (maternal country of destination, infant sex, maternal age groups, marital status, number of previous births, and father born in the same country as the mother), along with their respective standard deviations and number of observations. These aggregate estimates of each country were then combined and analyzed using regression techniques for aggregate data[27] to obtain unadjusted and adjusted mean birthweight differences and odds ratios with 95% confidence intervals, using births occurred in Spain as the reference group. Regression analysis of continuous and categorical aggregate data produces point estimates and confidence intervals similar to those obtained with individual-level data.[27] To account for unmeasured potential variations in circumstances of health care between receiving countries (i.e., geographic setting, health care setting, type of health care providers, and differences in the medical management between comparison groups) we further adjusted for a comparability score[28] by assigning a weight of 1/8 to each observation of the reference group (i.e., women delivering in Spain). Regression analyses were adjusted for infant sex, maternal age groups, marital status, number of previous births, and father born in the same country as the mother, as these factors may act as potential confounders.

Finally, we conducted secondary analyses restricted to Ontario hospital data linked to the official immigration register (Citizenship and Immigration Canada Database) to assess whether time since arrival to Canada was associated with improved birth outcomes of Latin Americans in Ontario, as a potential alternative explanation to our findings.

## Results

The share of singleton births to Latin American immigrant women was larger in Spain (4.56%) than in Canada (2.18%) (Table 1). Latin American women delivering in Spain were more likely to be teenagers, unmarried and not having delivered previously. They were also more likely to have a partner born in the same country where they were born.

**Table 1:** Characteristics of the study populations*.

	Canada (2000–2005)		Spain (1998–2007)	
	Native-born	Latin America-born	Native-born	Latin America-born
**No of Singleton births**	1,427,351 (97.82)	31,767 (2.18)	3,145,891 (95.44)	150,405 (4.56)
**Infant sex (male)**	731,173 (51.2)	16,504 (52.0)	1,622,939 (51.59)	77,323 (51.41)
**Maternal age**				
< 20 years	81,069 (5.7)	1553 (4.9)	77,200 (2.45)	10,239 (6.81)
20–34 years	1,140,300 (79.9)	24,517 (77.2)	2,360,024 (75.02)	119,255 (79.29)
≥ 35 years	205,982 (14.4)	5697 (17.9)	708,667 (22.53)	20,911 (13.90)
**Marital status**			**-**	**-**
Married	809,104 (56.7)	24,112 (75.9)	2,501,359 (79.51)	64,049 (42.58)
Not married	474,162 (33.2)	5018 (15.8)	644,532 (20.49)	86,356 (57.42)
Unknown	144,085 (10.1)	2637 (8.3)	-	-
**Number of previous births**			** **	** **
None	648,818 (45.5)	12,633 (39.8)	1,742,653 (55.39)	82,246 (54.68)
1 or 2	681,760 (47.8)	15,095 (47.5)	1,343,001 (42.69)	61,234 (40.71)
≥ 3	96,773 (6.8)	4039 (12.7)	60,237 (1.91)	6925 (4.60)
Unknown				
**Gestational age, weeks**			** **	** **
20–31	11,406 (0.8)	264 (0.8)	16,917 (0.54)	1243 (0.83)
32–36	77,070 (5.4)	1526 (4.8)	177,453 (5.64)	8767 (5.83)
37–41	132,735 (9.3)	29,652 (93.3)	2,828,380 (89.91)	133,363 (88.67)
42–45	13,140 (0.9)	325 (1.0)	123,141 (3.91)	7032 (4.68)
**Birthweight, grams**			** **	** **
250–1499	9465 (0.7)	225 (0.7)	15,527 (0.49)	987 (0.66)
1500–2499	49,425 (3.5)	1029 (3.2)	156,497 (4.97)	6299 (4.19)
2500–3999	1,159,543 (81.2)	26,655 (83.9)	2,811,552 (89.37)	130,963 (87.07)
4000–5999	208,918 (14.6)	3858 (12.1)	162,315 (5.16)	12,156 (8.09)
**Father same country of birth**				
Same	1,229,047 (86.1)	14,943 (47.0)	3,031,060 (96.35)	86,465 (57.49)
Not the same	111,830 (7.8)	15,620 (49.2)	77,440 (2.46)	56,350 (37.46)
Unknown	86,474 (6.1)	1564 (4.9)	37,391 (1.19)	7590 (5.05)

* All data are expressed as number (%)

**Fig 1:**
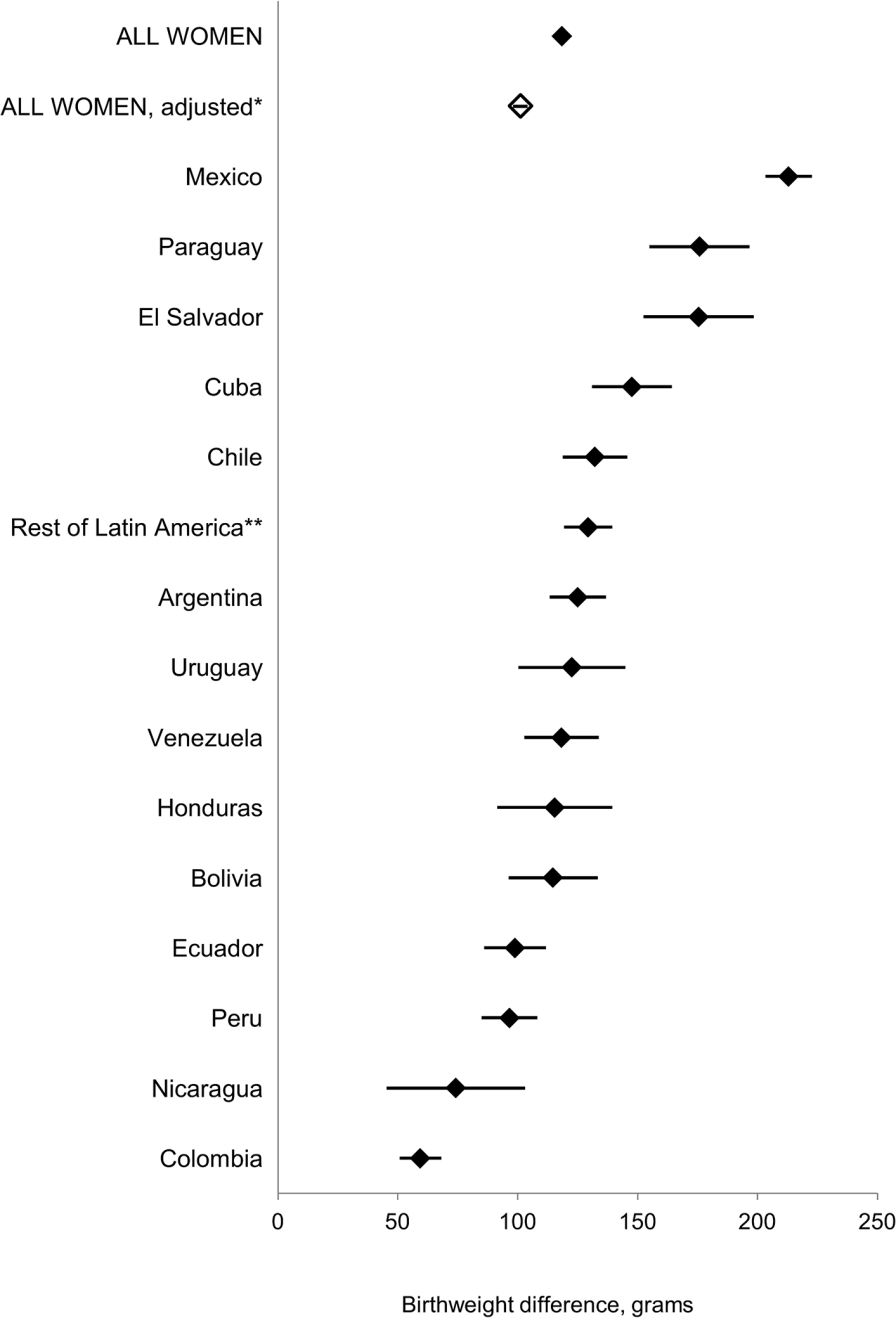
Mean birthweight differences (and 95% confidence intervals) between singleton infants born at 37–41 weeks of gestation to women from various Latin American countries who emigrated to and delivered in Canada (2000–2005) vs. those who emigrated to and delivered in Spain (1998–2007). * Adjusted for infant sex, maternal age groups (<20 years, 20–34, 35 or more), parity (primiparous, multiparous), married (yes, no), maternal country of birth same as father’s (yes, no). ** Rest of Latin America includes Costa Rica, Dominican Republic, Guatemala and Panama.

The mean birthweight at term was significantly higher for infants born to Latin American women residing in Canada compared to their counterparts residing in Spain, before (118 g, 95% CI: 115, 121) and after (101 g, 95% CI: 98, 104) adjustment (Fig 1). Further adjustment for comparability scores did not change the association (95 g, 95% CI: 91, 99). The Canadian advantage held true for each single maternal country of birth, the differences being larger for Mexicans (212 g, 95% CI: 203, 222) and less pronounced for Colombian women (59 g, 95% CI: 50, 67), with most countries in the range of 100 to 150 grams difference (Fig 1).

The rates of low birthweight and preterm birth were lower among infants born to Latin American women in Canada compared to those born to Latin American women in Spain (Table 2). The adjusted odds ratios were 0.88 (95% CI: 0.82, 0.94) for low birthweight and 0.88 (95% CI: 0.84, 0.93) for preterm birth, respectively.

**Table 2:** Singleton Low Birthweight (LBW) and Preterm birth (PTB) among native-born and Latin American women delivering in Canada vs. those delivering in Spain, overall and by maternal country of birth.

	Maternal country of delivery							
	Canada			Spain			Canada vs. Spain	
Maternal country of birth	Number of births	LBW(%)	PTB(%)	Number of births	LBW(%)	PTB(%)	LBWOdds Ratio (95% CI)	PTBOdds Ratio (95% CI)
Native-born	1,427,351	4.13	6.20	3,145,891	5.47	6.18	0.74 (0.73, 0.75)	1.00 (0.99, 1.01)
							0.70 (0.69, 0.71) ^b^	0.96 (0.95, 0.97) ^b^
All Latin American women	31,767	3.95	5.63	149,827	4.84	6.60	0.80 (0.75, 0.85)	0.84 (0.80, 0.89)
							0.88 (0.82, 0.94) ^b^	0.88 (0.84, 0.93) ^b^
***Latin American women*, *by country of birth***								
Argentina	1623	4.68	5.85	12,095	4.75	6.11	0.98 (0.77, 1.25)	0.95 (0.76, 1.19)
Bolivia	608	3.29	6.25	13,379	3.96	7.70	0.82 (0.52, 1.29)	0.79 (0.57, 1.11)
Chile	1986	3.27	5.49	2797	4.47	6.04	0.72 (0.53, 0.98)	0.90 (0.70, 1.15)
Colombia	2731	3.81	5.35	30,007	4.72	6.76	0.79 (0.65, 0.97)	0.77 (0.65, 0.92)
Cuba	911	2.31	5.60	5250	4.30	5.41	0.52 (0.33, 0.82)	1.03 (0.76, 1.40)
Ecuador	1303	4.91	6.91	56,721	5.04	6.67	0.97 (0.75, 1.25)	1.03 (0.83, 1.29)
El Salvador	4237	5.52	7.84	354	6.50	6.50	0.84 (0.54, 1.30)	1.22 (0.79, 1.89)
Honduras	565	4.42	5.66	937	5.34	6.72	0.82 (0.50, 1.34)	0.83 (0.53, 1.29)
Mexico	9780	3.21	4.53	2435	4.27	4.89	0.74 (0.59, 0.93)	0.92 (0.75, 1.13)
Nicaragua	1049	4.67	6.01	325	5.85	7.38	0.78 (0.45, 1.36)	0.80 (0.49, 1.30)
Paraguay	615	3.58	3.41	2570	5.06	7.39	0.69 (0.43, 1.10)	0.44 (0.27, 0.70)
Peru	1869	3.48	4.76	8821	4.21	6.21	0.82 (0.62, 1.07)	0.75 (0.59, 0.94)
Uruguay	459	5.66	6.97	3082	5.48	5.45	1.03 (0.67, 1.58)	1.29 (0.87, 1.92)
Venezuela	1035	3.29	4.64	4190	6.56	6.35	0.64 (0.44, 0.92)	0.71 (0.52, 0.98)
Rest of Latin America ^a^	2996	4.51	6.71	6864	5.48	8.42	0.68 (0.56, 0.83)	0.78 (0.66, 0.92)

^a^ Includes Costa Rica, Dominican Republic, Guatemala and Panama

^b^ Adjusted for infant sex, maternal age groups (<20 years, 20–34, 35 or more), parity (primiparous, multiparous), married (yes, no), maternal country of birth same as father’s (yes, no)

Secondary analyses using the linked Ontario hospital birth records to the Canadian immigration register showed that time from arrival to delivery was not associated with increases in mean birthweight at term (Table 3). Low birthweight and preterm birth were only positively associated with length of stay in Canada among immigrants from the Andean region of South America. To assess comparability between the Ontario sub-sample of hospital births and the national Canadian data, we compared the study outcomes between Ontario and the rest of the country but there were no significant differences. In addition, in the Ontario data the study outcomes did not differ between refugees and non-refugee immigrants (data not shown).

**Table 3:** Changes in mean birthweight, low birthweight and preterm birth associated with a 10-year increase in length of residence among Latin American immigrant women giving birth in Ontario hospitals, Canada (1998–2007).

Maternal subregion of birth ^a^				Mean Birthweight at 37–41 weeks	Low Birthweight	Preterm Birth
	Number of births	Refugee (%)	Mean length of residence (SD)	10-year Mean difference, grams (95% CI) ^b^	10-year Odds Ratio (95% CI) ^b^	10-year Odds Ratio (95% CI) ^b^
Andean region	3809	21.0	5.5 (5.0)	8 (-39, 55)	1.65 (1.08, 2.53)	2.07 (1.31, 3.26)
Central America & Caribbean	9016	30.4	7.3 (5.4)	0 (-30, 29)	1.03 (0.80, 1.32)	1.24 (0.93, 1.65)
South Cone	1603	16.7	7.2 (5.6)	-7 (-76, 61)	0.79 (0.45, 1.42)	0.79 (0.42, 1.47)
All Latin American women	14,428	26.4	6.8 (5.4)	0 (-22, 23)	1.14 (0.94, 1.38)	1.30 (1.05, 1.61)

^a^ Maternal subregions of birth include Andean region (Bolivia, Colombia, Ecuador, Peru), Central America & Caribbean (Mexico, Cuba, Costa Rica, Dominican Republic, El Salvador, Guatemala, Honduras, Nicaragua, Panama, Venezuela), and South Cone (Argentina, Chile, Uruguay, Paraguay)

^b^ Adjusted for maternal age, maternal age^2, infant sex, neighborhood income quintiles, refugee status, marital status and maternal education at migration.

## Discussion

### Main findings

Our findings provide support to the hypothesis that selective migration may influence the more favourable birth outcomes of Latin American women delivering in Canada vs. in Spain. First, the share of singleton births to Latin American immigrant women was larger in Spain (4.56%) than in Canada (2.18%), despite the physical proximity of Latin America to Canada. Second, compared to Latin American women giving birth in Canada, those delivering in Spain were more likely to be teenagers, unmarried and not having delivered previously; these being correlates of adverse outcomes and factors associated with negative selection. Adjustment for these factors reduced the differences but did not eliminate them. Third and more important, birth outcomes were more favourable among Latin American women delivering in Canada vs. those delivering in Spain. Notably, mean birthweight at term was 59 to 212 grams higher among infants born to foreign-born women from different Latin American countries giving birth in Canada compared to those of their same-country counterparts giving birth in Spain. The magnitude of these differences is comparable to the effect of smoking on birthweight.[29,30]

### Strengths and limitations

Strengths of our study include the use of birth certificate data at the national level, comparable study periods and similar definition of the outcomes, maternal country of birth and covariates for statistical adjustment. Restriction of the study to a distinct immigrant group in two different receiving countries may diminish confounding resulting from unmeasured factors, since for example Mexican-born women giving birth in Canada may share background and cultural characteristics with their Mexican counterparts giving birth in Spain.

Limitations are several. First, gestational age information in Spanish birth certificates may slightly overestimate preterm birth rates, and consequently, low birthweight rates, according to a validation study that linked about 9000 birth certificates with records of one Madrilean hospital.[26] If this study was representative of Spain as a whole, then our findings regarding lower preterm birth and low birthweight rates among Latin American women delivering in Canada than in Spain should be taken with caution, since the true rates in Spain would be slightly lower, thus weakening the observed associations. This potential bias, however, does not affect our comparisons regarding birthweight at term. In the Madrilean study, the birthweight distribution at ≥37 weeks based on birth certificates was shown to be virtually identical to that of hospital records,[26] suggesting that the ubiquitous pattern of higher mean birthweight at term found among women from every Latin American country giving birth in Canada compared to Spain is unlikely to be affected by artifacts. Misclassification of gestational age in birth certificates is known to affect preterm and post-term births but not term births.[31,32]

While low birthweight and preterm birth mainly reflect pathological processes interrupting the pregnancy or its normal development, mean birthweight at term is a measure of normal fetal growth in the population.[33] If there were no significant differences in low birthweight and preterm birth rates between Latin American women delivering in Canada versus those delivering in Spain, we can still conclude that selective migration is associated with improved fetal growth.

Second, although we controlled for some maternal characteristics associated with birth outcomes, birth certificates do not provide information on other potentially relevant information, such as pre-migration characteristics (e.g. education, occupation status, work experience, and social class in their home countries), health behaviors during pregnancy, and post-migration social position and health care use. To compensate, we further adjusted for comparability scores[28] designed to account for unmeasured characteristics of the study settings and the results remained statistically significant.

Third, the lack of information on the type of migrants and the motivations for migration hampers our ability to explore in greater depth the nature of selective migration in our main analyses. Our a priori hypothesis assumed a pattern based on legal and voluntary migration. While most immigrants fall within this group, many immigrants may also be refugees or unauthorized. Births to both refugees and unauthorized immigrant mothers are captured, however, in both Canadian and Spanish birth certificates but it was not possible to identify them to conduct sub-group analyses. In the Ontario sub-sample, however, information on refugee status was available but refugee status was not associated with the study outcomes. The Ontario cohort was restricted to legal immigrants to Canada and therefore could not shed light on whether birth outcomes are different between legal and undocumented migrants.

Finally, we could not provide a more complete picture of the potential effects of selective migration on birth outcomes by including data of Latin American women who did not migrate and gave birth in their home countries.

### Significance of the study

To our knowledge, this is the first study focusing on the impact of selective migration on birth outcomes in relation to the immigrant admission context of the receiving countries. A recent study comparing preterm birth rates of Mexico-born women delivering in Mexico and in California did not find evidence of a healthy immigrant effect.[10] This study, however, could not differentiate between authorized and unauthorized migrants, who are numerous in California, and therefore whether the healthy migrant effect applies differently to these two groups. A study conducted in New York City suggested that undocumented foreign-born Latinas had higher low birthweight rates than documented foreign-born Latinas.[34]

Our findings suggest that characteristics of the immigrant-receiving countries may be associated with differential selective migration patterns, which in turn may translate into different health outcomes. More specifically, the more restrictive immigration admission context in Canada seems to produce a “cream skimming” effect, further selecting women with characteristics conducive to favourable birth outcomes beyond self-selection to migrate. Selective migration of Latin Americans may take place both in Spain and Canada, but our findings suggest that it is stronger in Canada. While self-selection to emigrate may result in better birth outcomes among those who emigrated compared to those who did not migrate, Canada’s more restricted admission policies place a second filter on those who self-select themselves for migration, thus boosting the selection effect.In addition, Spain’s Latin American immigrant population has grown since the 2000s partly because of high demand for low-skill temporary laborers, many of whom became permanent residents through the 2005 regularization program. In Canada, there has not been a similar strong pull factor attracting lower SES Latin American immigrants.

While the selection hypothesis places emphasis on pre-migration characteristics, a potential alternative explanation to our findings is that the observed differences may reflect a more favourable post-migration socioeconomic environment in Canada than in Spain. While most Latin American women giving birth in Spain were recent immigrants, since immigration to Spain is a recent phenomenon that achieved its peak during the study period,[35] immigration to Canada has been more stable, meaning that Latin Americans have spent a longer time from arrival to the time of giving birth there. If the higher birthweight of Latin Americans in Canada is a result of adaptation to the local environment and improved living conditions, we would expect an increase in mean birthweight with longer stay in Canada. However, our analyses based on the Ontario sub-sample do not support that hypothesis. Prolonged time from migration to delivery was not associated with better outcomes thus contributing to a relative advantage of Canada versus Spain. Indeed, preterm birth increased rather than decreased with longer stay in Canada, as observed in other immigrant studies,[36,37] which could have contributed to a dilution of the cross-national differences. A longer residence of Latin American women in Canada does not explain our results.

Differences in the proportion of refugees in Canada and in Spain are unlikely to contribute to explain the observed differences. In our sensitivity analyses restricted to Ontario data, we did not find an association between refugee status and birth outcomes. Refugees to Spain are very few[20] and unlikely to influence birth outcomes of Latin Americans in Spain.

Finally, the presence of unauthorized immigrants may play an important role in explaining our findings. Although the unauthorized immigrant population in Canada is small,[18] it constitutes a sizable proportion of the overall immigrant population in Spain[21,22] and an unknown proportion of Latin American women giving birth in Spain during the study period may have stayed without governmental permission. In Spain, unauthorized residents have rights to healthcare during pregnancy, partum and postpartum. It was estimated that unauthorized stay accounts for about 25% of female immigrant workers aged 20 to 40 years in Spain in 2008.[38] Unauthorized immigrants may be negatively selected in terms of human capital and may experience socioeconomic disadvantage, challenges with healthcare access, discrimination, and fear of deportation in the receiving country;[39,40] stressors which may contribute to poor birth outcomes and therefore eroding any potential advantage associated with positive self-selection.

In sum, our findings provide support for the selective migration hypothesis, particularly due to a stricter immigrant selection in Canada compared to Spain. While most research on immigrant health focuses on the characteristics of the immigrant populations in relation to their places of origin, this study highlights the importance of paying attention to the destination environments, particularly the combination of “push-pull’ factors may determine the health trajectories of newly arrived immigrants. Future studies focusing on the differences in health outcomes between authorized and unauthorized migrants may help clarify the roles of self- and state-selection and the healthy migrant effect.
